# Genetic Evaluation of Beef Sires Using a Beef‐on‐Dairy Crossbred Reference Population

**DOI:** 10.1111/jbg.70030

**Published:** 2025-11-15

**Authors:** Julius Mugambe, Christin Schmidtmann, Jorge Hidalgo, Rana Ahmed, Georg Thaller

**Affiliations:** ^1^ Department of Animal Biosciences, SLU Interbull Centre Uppsala Sweden; ^2^ Institut für Tierzucht Und Tierhaltung Christian‐Albrechts‐Universität Zu Kiel Kiel Germany; ^3^ IT‐Solutions for Animal Production (Vit) Verden Germany; ^4^ Department of Animal and Dairy Science University of Georgia Athens Georgia USA

**Keywords:** beef‐on‐dairy, birthweight, calving ease, threshold‐linear models

## Abstract

In recent years, Beef‐on‐Dairy (BoD) crossbreeding programs have gained momentum to enhance dairy cattle's economic and genetic merits while meeting the demand for high‐quality beef. However, bulls with superior growth potential can lead to calving problems; thus, Holstein dairy farmers must decide which semen to use to avoid calving problems while producing heavier BoD calves. In this study, our objective was to genetically evaluate beef sires using a BoD crossbred reference population for three major economic traits, i.e., gestation length (GL), birth weight (BW), and calving ease (CE). A population comprising 4420 BoD calves sired by bulls from Angus (ANG), Limousin (LIM), Wagyu (WAG), and White‐Belgian Blue (WBB) was used to perform a joint genetic evaluation of the sire for traits. Univariate and bivariate (linear‐linear or threshold‐linear) models were applied to estimate variance components and genomic breeding values using single‐step methods. Estimates from CE models were transformed from the liability to the observable scale for more straightforward interpretation. Direct heritabilities for GL, BW, and CE (after transformations) ranged from 0.33 to 0.35, 0.33 to 0.37, and 0.02 to 0.14, respectively, while maternal heritabilities ranged from 0.11 to 0.17 for all traits. Generally, male BoD calves had higher probabilities for calving difficulty, with calvings being more difficult if sired by LIM (13%) as compared to ANG (7%) and WBB (9%) when considering male calves. The bivariate models outperformed the univariate models. For CE, the accuracy of predictions was up by 95% with a reduction in bias and dispersion. WBB sires were preferred when crossing with higher parity cows compared to ANG sires. These findings demonstrate that a well‐structured BoD reference population enables accurate genomic evaluation of beef sires, facilitating the selection of sires that produce economically viable calves with reduced calving difficulties.

## 1 Introduction

The dairy industry plays a vital role in global food production, providing a significant source of milk and dairy products (Blaskó 2011). However, the economic sustainability of dairy operations often faces challenges due to fluctuating milk prices and production costs. In response to these challenges, “Beef‐on‐Dairy” (BoD) crossbreeding programs have emerged as a promising approach to enhance the profitability of dairy enterprises while simultaneously producing BoD calves with desirable traits for meat production, including carcass quality and terminal performance (Berry 2021; Mota et al. 2022; Berry et al. 2023). BoD leverages the superior meat quality and growth potential of beef bulls, but their use can lead to prolonged gestation length (GL) and increased calving difficulties, impacting cow health and farm economics (Fouz et al. 2013; Eriksson et al. 2020). A previous study indicated that calving difficulties occurring in approximately 10%–15% of dairy cow births could lead to average economic losses estimated at €125–€243 per incident due to veterinary costs, reduced milk yield, and calf mortality (Martin‐Collado et al. 2017). Genetic evaluations focusing on calving ease (CE), birth weight (BW), and GL can mitigate these issues (Eaglen et al. 2012; Purfield et al. 2020).

Sire breed influences calf traits and dam lactation performance and health, with some studies noting increased dystocia risks with certain beef breeds (Basiel et al. 2024). However, other research suggests no significant impact on cow health or milk yield (Basiel et al. 2024), highlighting the need for precise sire selection. To mitigate these challenges, genomic evaluations must consider the complexities of crossbreeding. Examination of the genetic effects of crossbreeding beef cattle highlighted the potential of utilizing bulls with specific genetic merit for traits like BW and GL to enhance CE and adjust gestation periods (Foraker et al. 2022; Coleman et al. 2023).

Genetic evaluations involving CE and BW are reported to solve difficult calvings (Eaglen et al. 2012; Ahlberg et al. 2016; Eriksson et al. 2020; Purfield et al. 2020). Thus, a joint genomic evaluation incorporating purebred and crossbred data improves accuracy when crossbreds are genotyped (Steyn et al. 2019; Misztal et al. 2022). However, crossbreeding introduces genetic complexity due to varying linkage disequilibrium (LD) patterns across breeds (Ibáñez‐Escriche et al. 2009). Unlike purebred evaluations, BoD‐specific evaluations should account for breed interactions, ensuring more relevant GEBVs for crossbred performance. Several methods have been proposed for genetic evaluations involving different breeds, given that the reference population includes the specific breed type(s) (Steyn et al. 2019; Khansefid et al. 2020; VanRaden et al. 2020; Eiríksson et al. 2021). A joint evaluation of all purebreds and crossbreds with a single additive effect has been proven to have the potential to improve accuracy, especially if the crossbreds are all genotyped (Steyn et al. 2019; Harris 2022; Misztal et al. 2022).

This study validates using a BoD crossbred reference population to evaluate GL, BW, and CE beef sires. Objectives included estimating variance components and GEBVs to guide sire selection for optimal BoD calf production. Additionally, we explored the potential of including carcass and terminal trait predictions in BoD evaluations to align with the growing beef‐on‐dairy market.

## 2 Materials and Methods

Animal care and use committee approvals were unnecessary because no animals were used in this study.

### 2.1 Animals and Data

The reference population in this study comprises records of 4420 BoD crossbred calves collected between 2017 and 2023 from 224 dairy farms in Schleswig‐Holstein, Germany.

All BoD calves were born to Holstein (HOL) dams and had sires from one of the following beef breeds: Angus (ANG), Limousin (LIM), Wagyu (WAG), and White‐Belgian Blue (WBB). The farmers owning these calves aimed solely at selling them to fattening farms through auctions, as in Dal Zotto et al. (2009). Crossbred calves that were heavier and possessed appealing aesthetic traits were sold at the highest prices. Three traits of interest were considered in this study, i.e., GL, BW, and CE, all considered to be traits of the calf.

GL (in days) was calculated as the difference between the dam's confirmed successful insemination date and the calf's birthdate. The dams used in the study had GLs ranging from 265 to 301 days.

The weight of the calves was measured once between 0 and 40 days of life, with around two‐thirds of the calves being younger than ten days at the time of weighing. All BoD calves were measured using a weighing scale in kilograms. Using the recorded weights of calves, BW was age‐adjusted for the calves that were not measured at day zero, as in Ahmed et al. (2024). The data‐cleaning process involved the removal of all calves without records or whose parentage was unknown.

The descriptive statistics of the data are shown in Table 1. GL and CE were measured on the cow but considered traits of the BoD calf.

**TABLE 1:** General statistics for gestation length (GL), birth weight (BW) and calving ease (CE).

Trait	Mean (SD)	Min	Max	BoD calves	Genotyped animals
GL	281.6 (5.0)	265.0	301.0	4420	5201
BW	48.2 (5.3)	23.2	71.2	3661	4838

		Incidence (%)	BoD calves	Genotyped animals
CE	Easy	89.0 11.0	3257	4838
	Difficult		404	

Abbreviations: BW, age‐adjusted birth weight (kg); CE, calving ease incidences (%); GL, gestation length (days).

**FIGURE 1:**
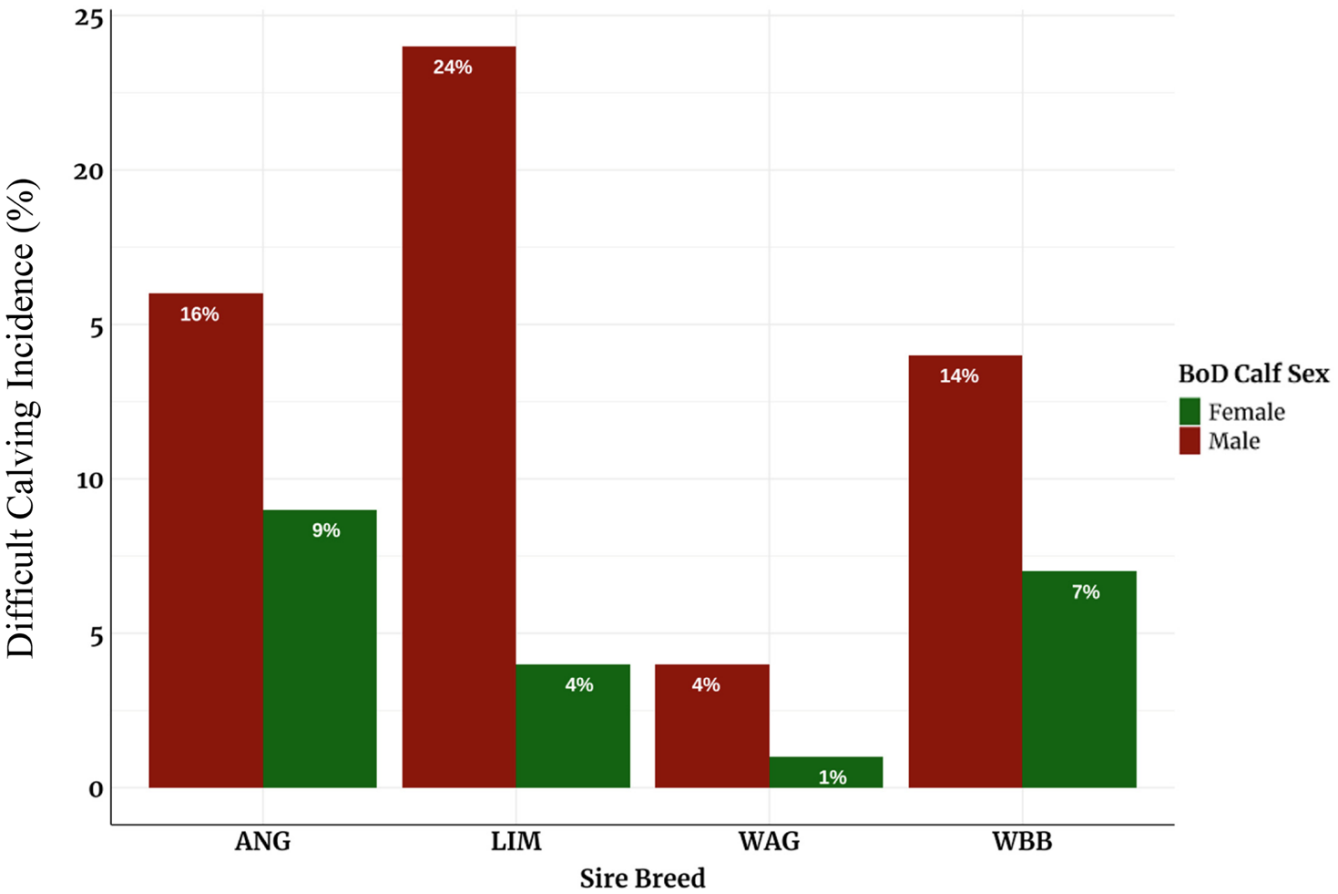
The difficult calving incidence (%) per sire breed according to the sex of the BoD calf. [Colour figure can be viewed at wileyonlinelibrary.com]

CE was recorded according to the guidelines of the International Committee of Animal Recording (ICAR 2022) with four distinct categories: (1) easy/unassisted, (2) easy pull, (3) difficult calving/hard pull, and (4) caesarean section/embryotomy. Due to a few records in some categories, CE was converted to a binary trait, where 1 = easy calving (combining 1 and 2) and 2 = difficult calving (combining 3 and 4). The incidence of assisted calving was 11% in the entire dataset. Figure 1 shows the incidences of CE per sire breed and according to the sex of the calf. The male BoD calves had higher incidences of difficulty calving than the female ones. Table 2 shows the descriptive statistics for the three traits according to sire breed.

**TABLE 2:** Descriptive statistics for the traits according to sire breed with standard deviations in parentheses.

Sire breed	No. sires	No. BOD calves	Mean parity	BW (kg)	GL (days)
ANG	11	832	2.2	45.4 (4.4)	280.2 (4.9)
LIM	5	177	3.4	45.4 (5.4)	288.1 (5.7)
WAG	8	151	2.5	42.7 (3.0)	283.0 (4.6)
WBB	33	2936	3.5	49.6 (4.9)	281.6 (4.8)

Abbreviations: ANG, angus; BW, age‐adjusted birth weight (kg); CE, calving ease incidences (%); GL, gestation length (days); LIM, limousin; WAG, Wagyu and WBB, Belgian Blue.

#### 2.1.1 Genotypes and Quality Control

For all BoD calves in the reference population, 50 k chip genotypes were available. Moreover, 1147 dams and 30 sires had genotype information. Quality control was performed using PREGSF90 software (Misztal et al. 2014), which included the elimination of duplicated genotypes, individuals, and SNPs with a call rate < 0.90, SNPs with a minor allele frequency < 0.05 or with a departure from Hardy–Weinberg equilibrium (difference between the observed and expected heterozygous frequency) > 0.15. Parent‐progeny pairs were tested for Mendelian conflicts; SNPs with a conflict rate > 10% were removed, and BoD calves were eliminated if the conflict rate was > 1% (as a percentage of all SNPs). After quality control, 42,897 autosomal markers and 4838 animals were kept for further analysis.

#### 2.1.2 Pedigree

The pedigree used in this study comprised 24,179 animals traced back three ancestral generations for the animals with phenotypic and genomic information. Out of the 30 genotyped sires, only 15 were in the pedigree alongside all the dams.

### 2.2 Statistical Analyses

#### 2.2.1 Variance Components

All variance components were estimated by fitting univariate (linear or threshold animal models) or bivariate (linear‐linear or threshold‐linear) models. We used single‐step genomic restricted maximum likelihood (ss‐GREML) in the linear and linear‐linear models. We used a Bayesian implementation through Gibbs sampling (ss‐Gibbs) when fitting threshold and linear‐threshold models.

#### 2.2.2 Univariate Models

##### 2.2.2.1 Birth Weight and Gestation Length

The following linear animal model was used for BW and GL:

(1) $$y=\mathrm{Xb}+Z_{1}a+Z_{2}m+Z_{3}s+e,$$

where **y** represents the vector of phenotypes (BW or GL); **b**, **a**, **m**, **s** and **e** are vectors of fixed effects (fixed effects: herd‐year‐season [427 levels], dam parity [1–9], sire breed [ANG/LIM/WAG/WBB], calf sex [male/female]), additive direct genetic effect, additive maternal genetic effect, the random sire and residual, respectively. **X**, $Z_{1}$, $Z_{2}$, and $Z_{3}$ are the incidence matrices for **b**, **a**, **m**, and **s**, respectively.

##### 2.2.2.2 Calving Ease

A threshold animal model was fit to analyse CE. The statistical model can be represented as in (1); however, the binary phenotype [**y** = {$y_{i}$}(*i* = 1, 2, …, *n*)] is assumed to be the response of an underlying liability ($l_{i}$), which, conditionally to the model parameters, follows a normal distribution with mean vector **Kθ** and unit variance:

(2) $$l|b,a,m,s,\sigma_{e}^{2}\sim N\left(\mathrm{K\theta},{I\sigma }_{e}^{2}\right),$$

where $\theta^{\prime }=\left(b^{\prime },a^{\prime },m^{\prime },s\right)$ is a vector of fixed and random effects and K is an incidence matrix linking θ to the phenotypic records.

The conditional response of the binary phenotypes was modeled with the following distribution:

(3) $$P\left(y|l,t\right)=\prod_{i=1}^{n}\left[I\left(l_{i}\leq t\right)P\left(y_{i}=1|l_{i},t\right)+\right.\left.I\left(l_{i}>t\right)P\left(y_{i}=2|l_{i},t\right)\right],$$

where $I\left(\cdot \right)$ is an indicator function that takes the value of 1 if the specified condition inside the parentheses is true; otherwise, it takes the value of 0, and t is the threshold.

#### 2.2.3 Bivariate Threshold‐Linear Models

When BW records are available, a bivariate threshold‐linear model is recommended as a better alternative to univariate threshold models in predicting both direct and maternal effects when analysing CE (Varona et al. 1999). The bivariate models took the form shown below:

(4) $$\left[\begin{array}{c} y_{1}\\ y_{2} \end{array}\right]=\left[\begin{array}{cc} x_{1} & 0\\ 0 & x_{2} \end{array}\right]\left[\begin{array}{c} b_{1}\\ b_{2} \end{array}\right]+\left[\begin{array}{cc} {z_{1}}_{t1} & 0\\ 0 & {z_{1}}_{t2} \end{array}\right]\left[\begin{array}{c} a_{1}\\ a_{2} \end{array}\right]+\left[\begin{array}{cc} {z_{2}}_{t1} & 0\\ 0 & {z_{2}}_{t2} \end{array}\right]\left[\begin{array}{c} m_{1}\\ m_{2} \end{array}\right]+\left[\begin{array}{cc} {z_{3}}_{t1} & 0\\ 0 & {z_{3}}_{t2} \end{array}\right]\left[\begin{array}{c} s_{t1}\\ s_{t2} \end{array}\right]+\left[\begin{array}{c} e_{1}\\ e_{2} \end{array}\right],$$

where vectors $y_{1}\;\text{and}\;y_{2}$ are the phenotypes for the trait combinations, i.e., BW_CE and GL_CE, respectively; **b, a, m, s** and **e** are the vectors of fixed effects (as described above), respectively. **X**, $Z_{1}$, $Z_{2}$ and $Z_{3}$ are the incidence matrices.

The vectors **a**, **m**, **s** and **e** were assumed to follow multivariate normal distributions, i.e., $\left[\begin{array}{c} a\\ m \end{array}\right]\sim N\left[0,\begin{array}{cc} V_{a}⨂H & V_{a,m}⨂H\\ V_{m,a}⨂H & V_{m}⨂H \end{array}\right]$, **s** ~ *N* (**0**, $V_{s}⨂I$), and **e** ~ *N* (**0**, $V_{e}⨂I$) where $V_{a}$, $V_{a,m}=V_{m,a}$, $V_{m},V_{s}$, and $V_{e}$ are 2×2 co(variances)matrices for the direct genetic, maternal genetic, random sire and random residual variances, respectively. **I** is an identity matrix of proper order, and **H** is the combined pedigree‐based and marker‐based relationship matrix defined as (Legarra et al. 2009; Christensen and Lund 2010):

(5) $$H=\left[\begin{array}{cc} A_{11}-A_{12}A_{22}^{-1}A_{21}+A_{12}A_{22}^{-1}{\mathrm{GA}}_{22}^{-1}A_{21} & A_{12}A_{22}^{-1}G\\ {\mathrm{GA}}_{22}^{-1}A_{21} & \left(1-\omega \right)G+\omega A_{22} \end{array}\right],$$

where ω is the blending factor, **A** is the pedigree relationship matrix, and **G** is the genomic relationship matrix constructed using VanRaden's first method (VanRaden 2008). The mean diagonals and off‐diagonals of **G** were adjusted (i.e., tuned) to those in the pedigree relationship matrix for genotyped animals ($A_{22}$) as described in Chen et al. (2011), to align both matrices to the same genetic base. Afterwards, to avoid singularity problems and account for a polygenic component explaining a proportion of the genetic variance unexplained by the genotypes (McWhorter et al. 2023), **G** was blended with 5% $A_{22}$ for all traits.

The phenotypic variance was obtained as:

(6) $$\sigma_{p}^{2}=\sigma_{a}^{2}+\sigma_{m}^{2}+\sigma_{\left(a,m\right)}+\sigma_{s}^{2}+\sigma_{e}^{2},$$

The bivariate models used are defined as ss‐GBLUP_GL_CE_, ss‐GBLUP_BW_CE_, and ss‐GBLUP_BW_GL_ for the three combinations of any two traits.

#### 2.2.4 Transformation of Variance Components

The variance components for CE estimated on the liability scale were transformed to the observed scale. The transformation was done according to Dempster and Lerner (1950).

(7) $$h_{\mathrm{obs}}^{2}=\frac{h_{\mathrm{lia}}^{2}z^{2}}{\left(p\left(1-p\right)\right)},$$

where $h_{\mathrm{obs}}^{2}$ is the heritability on the observed scale, $h_{\mathrm{lia}}^{2}$ is the heritability on the liability scale, p is the proportion of observations in one category and *z* is the height (probability density) of the ordinate of a standard normal distribution density function at the point corresponding to the threshold between the categories. This formula allows us to obtain the additive [direct ($\sigma_{a}^{2}$) and maternal ($\sigma_{m}^{2}$)], as well as the phenotypic ($\sigma_{p}^{2}$) variances on the observed scale. The random sire variance ($\sigma_{s}^{2}$) on the observed scale was estimated based on its relative contribution to the phenotypic variance on the liability scale. The residual variance on the observed scale was $\sigma_{e}^{2}$ = $\sigma_{p}^{2}$ −($\sigma_{a}^{2}+\sigma_{m}^{2}+\sigma_{\left(a,m\right)}+\sigma_{s}^{2}$). Genetic correlations within and across traits are invariant to changes in scale; therefore, those genetic parameters were kept constant.

#### 2.2.5 Transformation of Genomic Estimated Breeding Value

All genomic estimated breeding values (GEBVs) were obtained using ss‐GBLUP and the models described before. The GEBVs for CE were transformed into probabilities using the formula below:

(8) $$P_{i}=1-\Phi \left(\frac{t-\left(X_{i}^{\prime }b+Z_{i}^{\prime }a\right)}{\sigma_{e}}\right),$$

where $P_{i}$ is the probability of calving difficulty for animal i, Φ is the standard cumulative distribution function, t is the threshold, $\sigma_{e}$ is the residual standard deviation, and **X, Z, b**, and **a** are as defined before.

#### 2.2.6 Accuracy, Bias, and Dispersion

We investigated the accuracy, bias, and dispersion of genomic predictions using the linear regression validation using the LR method (Legarra and Reverter 2018). The LR method provides estimators to assess predicted breeding values based on partial (${\hat{u}}_{p}$) and whole datasets (${\hat{u}}_{w}$). In the whole dataset, we used all the available information, which represented the benchmark. The sires with at least 20 BoD calves were considered the focal/validation animals (see Table S1). Thus, in the partial dataset, we removed the phenotypes of these BoD calves. Estimators of accuracy, bias, and dispersion were obtained as described in Legarra and Reverter (2018). The accuracies of GEBVs using the LR method (${\hat{\mathrm{Acc}}}_{\mathrm{LR}}$) were calculated as follows:

(9) $${\hat{\mathrm{Acc}}}_{\mathrm{LR}}=\frac{\mathrm{cov}\left({\hat{u}}_{w},{\hat{u}}_{p}\right)}{\sigma_{a,i}^{2}}$$

where $\mathrm{cov}\left({\hat{u}}_{w},{\hat{u}}_{p}\right)$ is the covariance between estimated breeding values (EBVs) obtained with the whole dataset and EBVs obtained with the partial dataset, and $\sigma_{a}^{2}$ is the additive genetic variance of the population for a given trait. This formula is more general and differs from the original formula by Legarra and Reverter (2018), which is applied when the animals of interest are representative of their generation (they are not yet selected) as used in Macedo et al. (2020).

(10) $$\text{bias}={\hat{\Delta }}_{\mathrm{pw}}=\frac{\bar{{\hat{u}}_{p}}-\bar{{\hat{u}}_{w}}}{\sigma_{a}},$$

(11) $$\text{dispersion}={\hat{b}}_{w,p}=\frac{\mathrm{cov}\left({\hat{u}}_{w},{\hat{u}}_{p}\right)}{\mathrm{var}\left({\hat{u}}_{p}\right)}$$

Statistics of bias and dispersion were used to evaluate the unbiasedness of the predictions; a bias value of 0 and a dispersion value of 1 are desired and indicate unbiased predictions. An estimate of bias less than zero indicates an underestimation of GEBV in the validation animals, while that greater than zero indicates an overestimation. Additionally, estimates of dispersion greater than 1 indicate an overestimation of GEBV in the validation animals, and those greater than 1 indicate an underestimation.

#### 2.2.7 Software

The variance components involving linear models were estimated using Blupf90+, while those that involved CE were estimated using a Gibbs sampler implemented in Gibbsf90+. For all the models involving threshold models, a total of 500,000 samples were generated during Gibbs sampling, of which 100,000 were discarded as burn‐in while storing every 10th sample. Post‐Gibbs analyses were done using postgibbf90 to summarise the samples and produce post‐Gibbs statistics. All programs used are part of the BLUPF90 suite (Misztal et al. 2014).

## 3 Results

### 3.1 Genetic Parameters

This study presents variance components estimated using a crossbred reference population involving BoD crossbred calves sired by different breeds.

Estimated and transformed genetic parameters (in the case of CE) for all traits are presented in Tables 3 and 4. Both univariate and bivariate models yielded similar and comparable direct heritabilities for both BW and GL. The proportion of phenotypic variance explained by the sire effect for BW and GL using the univariate model was 0.11 and 0.12, while those from the bivariate models ranged from 0.12 to 0.13 for BW and from 0.09 to 0.15 for GL, respectively.

**TABLE 3:** Variance components from univariate models including transformed estimates for calving ease (CE) with standard errors in parentheses.

Trait	$\sigma_{a}^{2}$	$\sigma_{s}^{2}$	$\sigma_{m}^{2}$	$\sigma_{p}^{2}$	$\sigma_{e}^{2}$	$h_{a}^{2}$	$h_{m}^{2}$	$s^{2}$
GL	7.99 (1.27)	2.52 (1.48)	3.16 (1.03)	22.95 (1.51)	10.81 (0.07)	0.35 (0.05)	0.14 (0.04)	0.11 (0.05)
BW	0.60 (0.09)	0.19 (0.11)	0.24 (0.08)	1.72 (0.11)	0.81 (0.07)	0.35 (0.05)	0.14 (0.04)	0.12 (0.05)
${\mathrm{CE}}_{\text{liab}}$	0.13 (0.55)	0.16 (0.16)	0.69 (0.28)	2.27 (0.47)	1.01 (0.03)	0.06 (0.11)	0.30 (0.08)	0.07 (0.05)
${\mathrm{CE}}_{\mathrm{obs}}$	0.002	0.007	0.011	0.098	0.074	0.021	0.109	0.007

Abbreviations: BW, age‐adjusted birth weight (kg); ${\mathrm{CE}}_{\text{liab}}$, calving ease estimates on the liability scale; ${\mathrm{CE}}_{\mathrm{obs}}$, calving ease estimates on the observable scale; GL, gestation length (days); $s^{2}$, proportion of the total variance explained by the random sire variance.

**TABLE 4:** Variance components from bivariate models including transform estimates for calving ease (CE) with standard errors in parentheses.

Model	Trait	$\sigma_{a}^{2}$	$\sigma_{s}^{2}$	$\sigma_{m}^{2}$	$\sigma_{p}^{2}$	$\sigma_{e}^{2}$	$h_{a}^{2}$	$h_{m}^{2}$	$s^{2}$
$\mathrm{ss}-{\text{GBLUP}}_{\mathrm{GL}\_\mathrm{CE}}$	GL	8.12 (1.38)	3.74 (1.42)	4.28 (0.85)	24.34 (1.52)	10.27 (0.78)	0.33 (0.06)	0.18 (0.05)	0.15 (0.03)
	${\mathrm{CE}}_{\mathrm{lia}}$	1.18 (0.43)	0.27 (0.17)	1.63 (0.31)	3.55 (0.44)	1.01 (0.03)	0.33 (0.11)	0.46 (0.06)	0.08 (0.07)
	${\mathrm{CE}}_{\mathrm{obs}}$	0.012	0.007	0.016	0.098	0.068	0.121	0.166	0.076
$\mathrm{ss}-{\text{GBLUP}}_{\mathrm{BW}\_\mathrm{CE}}$	BW	0.78 (0.10)	0.35 (0.10)	0.25 (0.05)	1.85 (0.11)	0.70 (0.06)	0.37 (0.06)	0.12 (0.03)	0.13 (0.05)
	${\mathrm{CE}}_{\mathrm{lia}}$	1.26 (0.12)	0.34 (0.13)	1.27 (0.28)	3.26 (0.37)	1.01 (0.03)	0.39 (0.08)	0.39 (0.06)	0.11 (0.08)
	${\mathrm{CE}}_{\mathrm{obs}}$	0.014	0.010	0.014	0.098	0.067	0.140	0.141	0.011
$\mathrm{ss}-{\text{GBLUP}}_{\mathrm{BW}\_\mathrm{GL}}$	BW	0.70 (0.13)	0.25 (0.12)	0.27 (0.08)	1.85 (0.11)	0.84 (0.06)	0.38 (0.06)	0.14 (0.05)	0.12 (0.05)
	GL	9.33 (1.76)	2.45 (1.25)	3.65 (1.13)	26.60 (1.52)	11.31 (0.75)	0.35 (0.06)	0.14 (0.05)	0.09 (0.05)

Abbreviations: BW, age‐adjusted birth weight (kg); ${\mathrm{CE}}_{\text{liab}}$, calving ease estimates on the liability scale; ${\mathrm{CE}}_{\mathrm{obs}}$, calving ease estimates on the observable scale; GL, gestation length (days); $s^{2}$, proportion of the total variance explained by the random sire variance.

The heritability estimates of CE on the liability scale were higher than those obtained after the transformation to the observable scale (Tables 3 and 4). The direct heritability estimates for CE on the liability scale ranged from 0.06 to 0.39 and from 0.02 to 0.14 on the observed scale. Significant changes were observed in the maternal heritability when the transformation was made for the bivariate models as compared to the univariate model. The estimates for maternal heritability from the bivariate models on the liability scale were 0.46 (ss‐GBLUP_GL‐CE_) and 0.39 (ssGBLUP_BW_CE_), which changed to 0.17 and 0.14 on the observable scale, respectively.

### 3.2 Genetic Correlations

Genetic covariances and correlations from bivariate models are presented in Table 5. Negative genetic correlations between direct and maternal effects were observed for BW and GL as −0.26 and −0.35, respectively. Those for CE were −0.49 and −0.35 for the models involving the two combinations of traits, respectively (ss‐GBLUP_GL_CE_, ss‐GBLUP_BW_CE_). The strongest correlations were observed for ss‐GBLUP_BW_GL_, −0.30 for BW, and −0.89 for GL.

**TABLE 5:** Genetic covariances and correlations from bivariate models with standard errors in parentheses.

ss‐GBLUP_BW_CE_		ss‐GBLUP_GL_CE_	ss‐GBLUP_BW_GL_		
$\sigma_{a_{\mathrm{bw}},a_{\mathrm{ce}}}$	0.24 (0.16)	$\sigma_{a_{\mathrm{gl}},a_{\mathrm{ce}}}$	0.93 (0.26)	$\sigma_{a_{\mathrm{bw}},a_{\mathrm{gl}}}$	0.70 (0.10)
$\sigma_{m_{\mathrm{bw}},m_{\mathrm{ce}}}$	0.09 (0.10)	$\sigma_{m_{\mathrm{gl}},m_{\mathrm{ce}}}$	0.39 (0.24)	$\sigma_{m_{\mathrm{bw}},m_{\mathrm{gl}}}$	0.20 (0.15)
$\sigma_{a_{\mathrm{bw}},m_{\mathrm{bw}}}$	−0.10 (0.09)	$\sigma_{a_{\mathrm{gl}},m_{\mathrm{gl}}}$	−2.06 (1.07)	$\sigma_{a_{\mathrm{bw}},m_{\mathrm{bw}}}$	−0.50 (0.30)
$\sigma_{a_{\mathrm{ce}},m_{\mathrm{ce}}}$	−0.62 (0.04)	$\sigma_{a_{\mathrm{ce}},m_{\mathrm{ce}}}$	−0.42 (0.35)	$\sigma_{a_{\mathrm{gl}},m_{\mathrm{gl}}}$	−0.62 (0.04)
$\sigma_{e_{\mathrm{bw}},e_{\mathrm{ce}}}$	0.00 (0.09)	$\sigma_{e_{\mathrm{gl}},e_{\mathrm{ce}}}$	−0.08 (0.26)	$\sigma_{e_{\mathrm{bw}},e_{\mathrm{gl}}}$	0.05 (0.10)
$r_{a_{\mathrm{bw}},a_{\mathrm{ce}}}$	0.28 (0.13)	$r_{a_{\mathrm{gl}},a_{\mathrm{ce}}}$	0.29 (0.13)	$r_{a_{\mathrm{bw}},a_{\mathrm{gl}}}$	0.32 (0.12)
$r_{m_{\mathrm{bw}},m_{\mathrm{ce}}}$	0.17 (0.10)	$r_{m_{\mathrm{gl}},m_{\mathrm{ce}}}$	0.15 (0.10)	$r_{m_{\mathrm{bw}},m_{\mathrm{gl}}}$	0.18 (0.11)
$r_{a_{\mathrm{bw}},m_{\mathrm{bw}}}$	−0.26 (0.09)	$r_{a_{\mathrm{gl}},m_{\mathrm{gl}}}$	−0.35 (0.09)	$r_{a_{\mathrm{bw}},m_{\mathrm{bw}}}$	−0.30 (0.10)
$r_{a_{\mathrm{ce}},m_{\mathrm{ce}}}$	−0.49 (0.04)	$r_{a_{\mathrm{ce}},m_{\mathrm{ce}}}$	−0.35 (0.04)	$r_{a_{\mathrm{gl}},m_{\mathrm{gl}}}$	−0.89 (0.04)
$r_{e_{\mathrm{bw}},e_{\mathrm{ce}}}$	0.00 (0.09)	$r_{e_{\mathrm{gl}},e_{\mathrm{ce}}}$	−0.02 (0.09)	$r_{e_{\mathrm{bw}},e_{\mathrm{gl}}}$	0.03 (0.08)

Abbreviations: σ, covariance; a, direct genetic; bw, age‐adjusted birthweight; ce, calving ease; e, residual; gl, gestation length; m, maternal genetic; *r*, correlation.

The residual correlation between BW and CE was 0.00, while between GL and CE was −0.02, and the highest recorded for GL was 0.03.

### 3.3 GEBVs and Probabilities

Table 6 presents the mean sire GEBVs and their standard deviations for the traits GL, BW and CE (on the liability scale) for the four sire breeds: ANG, LIM, WAG and WBB. GEBVs were estimated using univariate and bivariate models, offering a comparative insight into the amount of variation or dispersion in the GEBVs estimated for a given sire breed.

**TABLE 6:** Mean sire GEBVs estimated from both bivariate and univariate models for the different sire breeds with standard deviations in parentheses.

Trait	Model	ANG	LIM	WAG	WBB
GL	Univariate	0.28 (0.15)	0.70 (0.07)	0.69 (0.08)	0.09 (0.07)
	Bivariate	−0.05 (0.30)	0.80 (1.05)	0.79 (0.99)	0.10 (0.29)
BW	Univariate	0.08 (0.32)	0.20 (0.54)	0.19 (0.30)	0.03 (0.30)
	Bivariate	0.09 (0.25)	0.24 (0.50)	0.24 (0.29)	0.07 (0.29)
CE	Univariate	0.06 (0.15)	0.07 (0.06)	0.08 (0.04)	0.07 (0.09)
	Bivariate	−0.17 (0.43)	0.21 (0.33)	0.14 (0.13)	0.02 (0.29)

Abbreviations: BW, age‐adjusted birth weight (kg); CE, calving ease (on liability scale); GL, gestation length (days).

Generally, LIM sires showed the highest GEBVs for the linear traits (BW and GL) from all models. For GL, the univariate model showed mean GEBVs ranging from 0.09 for WBB sires to 0.70 for LIM sires. Standard deviations were notably high for LIM sires for all models, indicating high variability among the LIM sires. From the bivariate models, the highest mean GEBVs were obtained from LIM and WAG for all three traits (Table 6).

**FIGURE 2:**
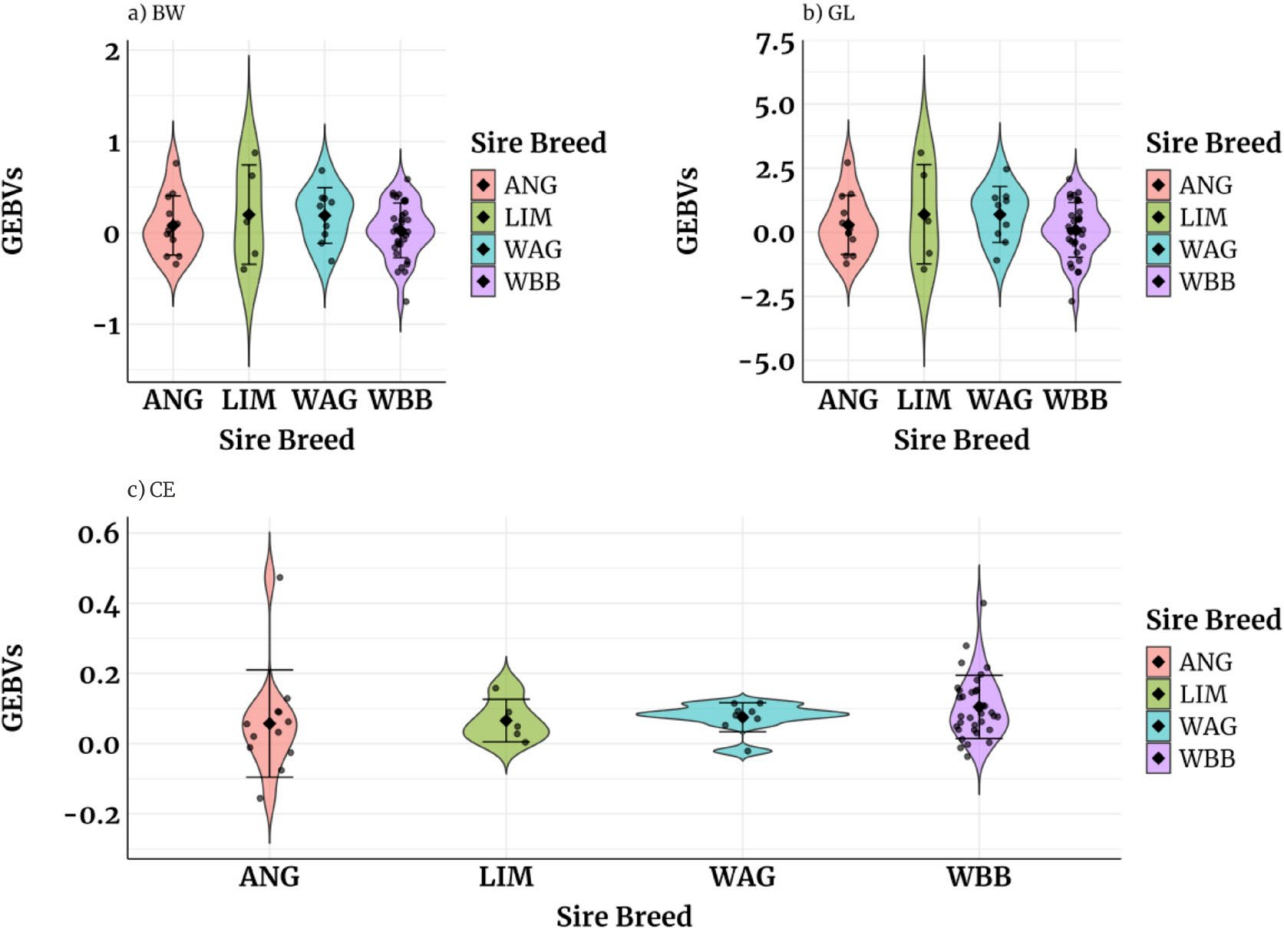
Violin plots showing estimated GEBVs for age‐adjusted BW (a), GL (b), and CE (c; on liability scale) using univariate models for the sire from different breeds. The violin midpoints indicate the mean GEBV. The whiskers represent the maximum and minimum GEBVs, and the dots represent the outliers. [Colour figure can be viewed at wileyonlinelibrary.com]

**FIGURE 3:**
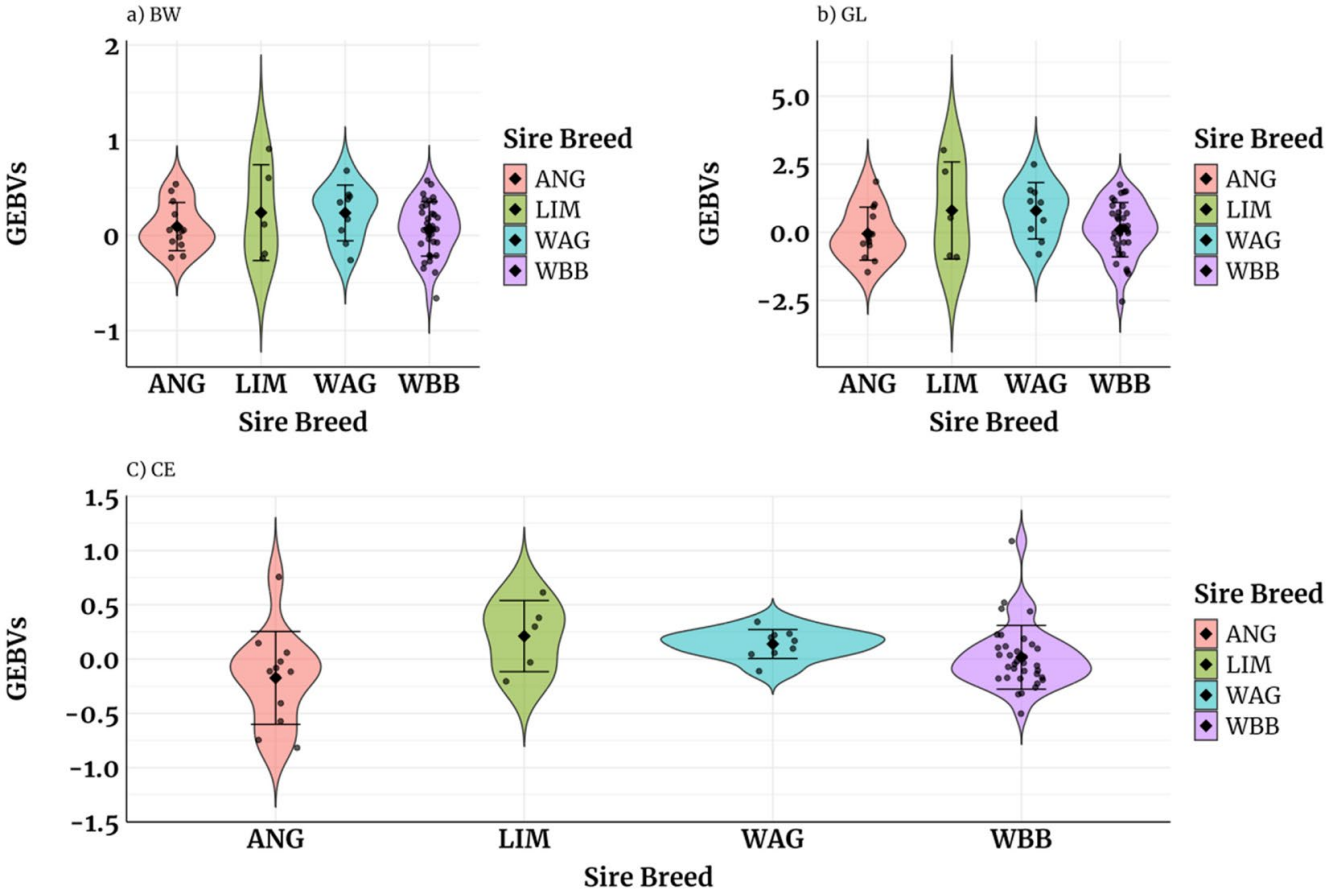
Violin plots showing estimated GEBVs for age‐adjusted BW (a), GL (b), and CE (c; on liability scale) using bivariate models for the sire from different breeds. The violin midpoints indicate the mean GEBV. The whiskers represent the maximum and minimum GEBVs, and the dots represent the outliers. [Colour figure can be viewed at wileyonlinelibrary.com]

**FIGURE 4:**
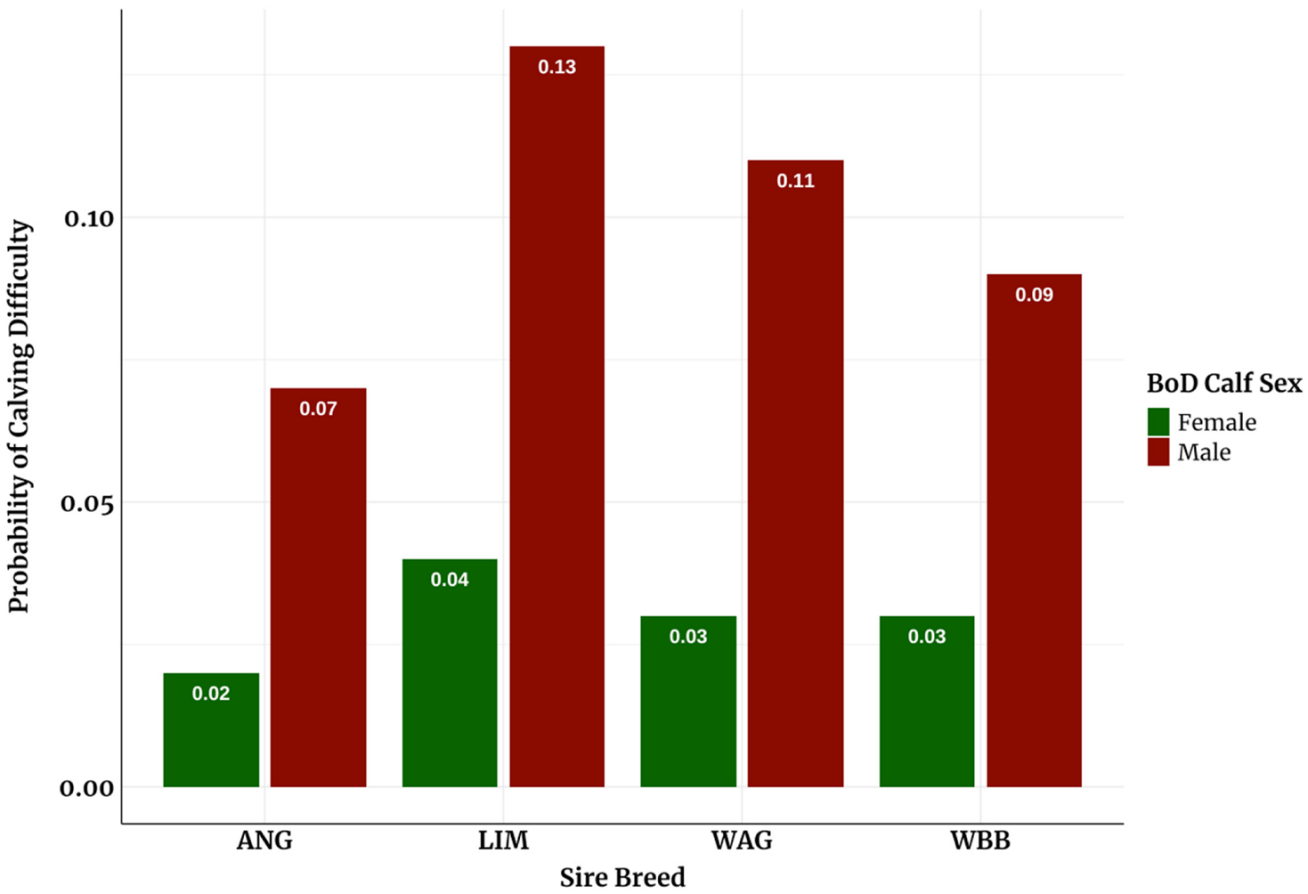
Mean probabilities of calving difficulty after transformation, categorized according to the sex of the BoD calf and the breed of the sire. [Colour figure can be viewed at wileyonlinelibrary.com]

The GEBVs presented in Table 6 for CE using both univariate and bivariate models are reported on the liability scale. Those from the univariate model ranged from 0.06 for ANG to 0.08 for WAG. Generally, the bivariate models for CE yielded higher mean GEBVs for LIM and WAG and the lowest for ANG sires. Figures 2 and 3 provide insights into the estimated GEBVs for all traits from both univariate and bivariate models. The probabilities associated with calving difficulty in Figure 4 are categorised based on the sex of the BoD calf. The results in our study show that the probability that calving will be difficult if the sex of the calf is male is higher than when it is female. LIM sires had the highest probability of difficult calving if the BoD calf was male (13%), while ANG sires had the lowest (7%). When the calf was female, only minor differences in the probability of difficult calving were observed between the different sires, ranging from 2% for ANG sires to 4% for LIM sires.

### 3.4 Accuracy, Bias, and Dispersion

**FIGURE 5:**
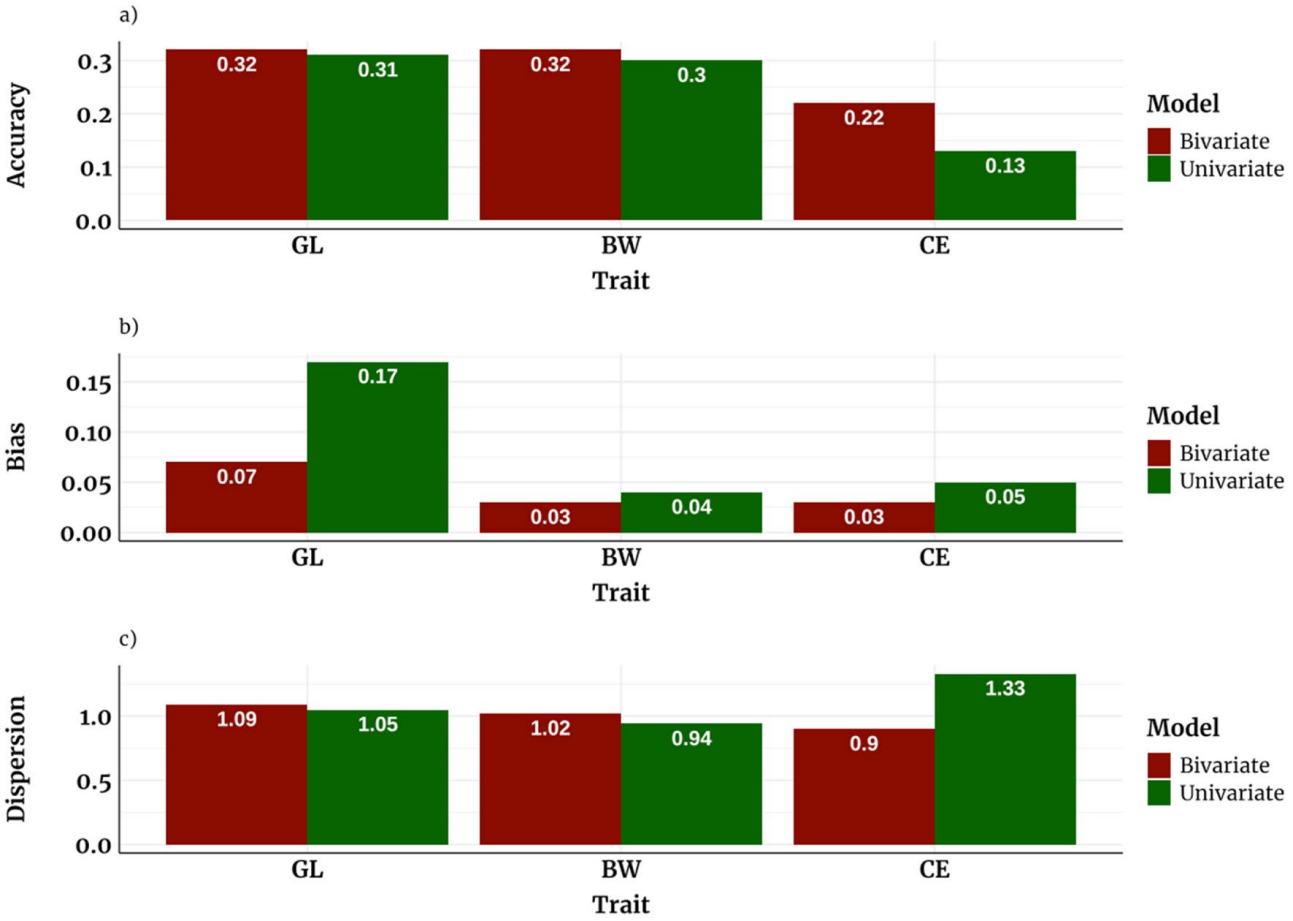
Accuracy, bias, and dispersion for three traits using the LR method: Gestation Length (GL), age‐adjusted Birth Weight (BW), and Calving Ease (CE) evaluated using univariate or bivariate models. [Colour figure can be viewed at wileyonlinelibrary.com]

The comparative analysis of accuracy, bias, and dispersion of GEBV for all traits and models is shown in Figure 5. Bivariate models yielded slightly higher values of accuracy for GL (0.32) and BW (0.32) compared to the univariate model (0.31 and 0.30, respectively). For CE, however, the bivariate model showed a notably higher accuracy (0.22) than the univariate model (0.13). This indicates that while both models performed similarly for GL and BW, the bivariate model demonstrated a significant improvement in accuracy for CE, which could be associated with the information shared across traits through genetic correlations.

The univariate model displayed a higher bias for GL (0.17) than the bivariate model (0.07). For BW (0.04 vs. 0.03) and CE (0.05 vs. 0.03), the univariate model showed negligible differences in bias compared to the bivariate model.

Slight differences were observed in the dispersion for GL and BW using both models. CE showed an under‐dispersion (the regression coefficient of whole on partial data = 1.33) when the univariate model was used, while the dispersion was much better using the bivariate model (dispersion = 0.90).

## 4 Discussion

The adoption of the use of beef semen on dairy cows requires sire selection to balance beef genetic potential with CE (Berry et al. 2020). Typically, semen from proven bulls selected for beef production can lead to calving difficulties in dairy cows. Previous studies have shown that calves sired by beef bulls had a higher risk of being stillborn, increased GL, and higher chances of difficult calving, thereby compromising animal welfare (Fouz et al. 2013; Eriksson et al. 2020; Basiel et al. 2024). Part of the solution involves estimating the breeding values of candidate sires while considering key traits. This study utilises a BoD crossbred reference population to estimate genetic parameters and evaluate the GEBVs of sires from various breeds.

### 4.1 Genetic Parameters

Even though numerous studies have estimated genetic parameters for GL, BW, and CE in purebred dairy or beef cattle (Varona et al. 1999; Mujibi and Crews 2009), few have focused on beef sires using a BoD crossbred reference population.

Heritabilities for the traits of interest have been observed to vary across breeds, with some comparable to others. For example, a recent study in Holsteins by Raschia et al. (2024) reported direct and maternal heritabilities for GL as 42% and 3%, respectively, while Norman et al. (2009) reported GL heritability estimates ranging from 10% to 47% across parity groups for seven US dairy breeds. In another study by Johanson et al. (2011), direct heritabilities of 26% and 51% were reported for BW and GL in a Holstein herd, with maternal heritabilities of 8% for both traits. In Japanese Black cattle, a heritability of 40% was reported for GL (Ibi et al. 2008). Charolais cattle, known for their robust physique, revealed a direct heritability of 46% for BW and 62% for GL (Mujibi and Crews 2009), while Nellore beef cattle had BW and GL heritabilities of 25% and 38%, respectively (Chud et al. 2014).

BoD heritabilities from our study (GL: 0.33–0.35; BW: 0.33–0.37; CE: 0.02–0.14 observable) blend dairy/beef genetics, potentially moderated by heterosis and LD differences (Ibáñez‐Escriche et al. 2009). Compared to purebreds (e.g., Holstein GL 0.42 direct, Raschia et al. 2024; Charolais BW 0.46, Mujibi and Crews 2009), BoD values are lower for CE, likely due to dairy dam constraints on beef calf size. WBB's higher BW/shorter GL vs. LIM (Table 2) reflects muscular hypertrophy/earlier maturity in WBB, vs. LIM's prolonged growth (Eriksson et al. 2020).

Heritability estimates for CE, when transformed to the observable scale, were lower (0.02–0.14) than those on the liability scale (0.06–0.39) due to the binary nature of the trait, as explained by Dempster and Lerner (1950). Genetic parameters for CE vary among cattle breeds due to complex genomic architecture and breed‐specific variability in calving performance. Compared to purebred dairy (8%–12% direct heritability; Vanderick et al. 2014) or beef (14%; Mujibi and Crews 2009), BoD CE heritabilities are lower, likely due to crossbred‐specific LD patterns and interactions between beef sire and dairy dam genomes. For example, in Walloon dairy cattle, direct heritability is approximately 8%–12%, with maternal heritability of 2%–4% (Vanderick et al. 2014, 2017). In Charolais, direct and maternal heritabilities for CE were 14% and 6%, respectively (Mujibi and Crews 2009). When threshold‐linear models were compared with linear‐linear models in simulated populations, direct heritabilities were 21% and 18%, with maternal heritabilities of 9% and 6%, respectively (Varona et al. 1999). In a Holstein herd, Johanson et al. (2011) reported direct and maternal heritabilities of 11% and 14%, respectively. These estimates align with our findings, emphasizing the importance of considering both direct and maternal effects in CE evaluations.

### 4.2 Genetic Correlations

In calving‐related traits, it is necessary to consider the correlations between direct and maternal genetic effects. In this study, negative genetic correlations were observed across all traits (e.g., −0.26 for BW, −0.35 for GL, −0.35 to −0.49 for CE), suggesting that selection for improved calf traits (e.g., heavier BW) may adversely impact dam traits (e.g., increased CE). This antagonistic relationship is stronger in BoD than in purebred dairy (−0.20 to −0.30; Vanderick et al. 2017) or beef (−0.15 to −0.25; Mujibi and Crews 2009), likely due to larger calf sizes from beef sires increasing dystocia risk in dairy dams (Fouz et al. 2013). The strong negative genetic correlation between BW and GL (−0.89, Table 5) indicates that sires producing heavier calves tend to have shorter GLs, beneficial for reducing calving difficulties. This also suggests that selection for shorter GLs in sires could inadvertently increase maternal genetic effects, potentially complicating calving outcomes due to mechanisms resembling genomic imprinting (Wolf and Hager 2008). This antagonistic relationship, more pronounced in BoD than in purebred dairy or beef populations (e.g., −0.35 for CE in Charolais, Mujibi and Crews 2009), may reflect the unique genetic architecture of crossbreds, where sire and dam contributions interact dynamically, necessitating careful balancing in breeding programs. However, the moderate negative direct–maternal CE correlations (−0.49 for BW_CE, −0.35 for GL_CE) highlight challenges in balancing calf size with maternal ease, necessitating careful sire selection to optimise both traits. Additionally, shared genetic pathways, possibly linked to fetal growth regulation genes, may be amplified in BoD due to beef sire contributions (Eriksson et al. 2020). Several studies have reported similar antagonistic relationships, with estimates comparable to ours (Varona et al. 1999; Ramirez‐Valverde et al. 2001; Eaglen et al. 2012; Vanderick et al. 2017).

Antagonistic direct‐maternal correlations (−0.26 BW, −0.35 GL, −0.35/−0.49 ce) suggest fetal‐maternal conflicts, where calf growth genes oppose maternal pelvis/fitness constraints (Wolf and Hager 2008). These may be inflated in BoD due to dairy‐beef mismatches, exacerbating CE antagonism vs. purebreds (e.g., −0.30 in Charolais, Mujibi and Crews 2009). Positive across‐trait correlations (e.g., 0.70 BW–GL) indicate shared growth pathways, but low residuals suggest environmental independence.

### 4.3 GEBVs and Probabilities

With estimated GEBVs, farmers can make informed mating decisions to maximise calf value and marketability in BoD programmes (Berry et al. 2020, 2023). In this study, variance components from univariate models were used to estimate GEBVs for linear traits (GL, BW), while bivariate models were used for CE to improve accuracy. The bivariate BW–GL model was also analysed (see Table 5), showing a strong genetic correlation (−0.32 for direct effects), reinforcing the value of multi‐trait models in capturing trait interactions.

Using both linear and threshold models, we utilised a BoD crossbred reference population to estimate GEBVs for sires of different breeds. Including 1147 genotyped dams and 30 genotyped sires alongside 4420 BoD calves enhanced CE accuracy by capturing maternal and direct effects (Steyn et al. 2019). While GEBVs from linear models showed a strong correlation (≥ 0.96) with those from threshold models, transformations to probabilities for CE (on the observable scale) facilitate easier interpretation. Probabilities were reported on the observable scale (Figure 4) to reflect practical outcomes, as liability‐scale GEBVs are less intuitive for decision‐making. For example, male BoD calves sired by LIM had a 13% probability of difficult calving compared to 7% for ANG, guiding sire selection based on calf sex and cow parity.

Results showed that sires with the highest positive GEBVs for CE on the liability scale (e.g., LIM: 0.21, WAG: 0.14) had the highest probabilities of difficult calving. ANG (−0.17) and WBB (0.02) sires showed the lowest probabilities, making them preferable for minimising calving issues. These findings align with Ahlberg et al. (2016), who found that breeds like Angus are less prone to calving assistance, while Limousin requires more. However, Eriksson et al. (2020) noted that selecting beef bulls with high EBVs for CE can reduce difficulties, even in later‐maturing breeds.

ANG sires that showed the lowest probability of difficult calving were primarily used on heifers (Table 2). In contrast, WBB sires were used on higher‐parity cows with slightly higher but manageable CE probabilities. This reflects a balance between minimizing calving risks and maximizing BW, as WBB‐sired calves were heavier (49.6 kg vs. 45.4 kg for LIM). Genomic predictions enable sire selection within breeds, identifying individuals with optimal traits (Figures 2 and 3).

To optimise the economic value of beef‐on‐dairy (BoD) calves, integrating carcass and terminal traits, such as marbling and muscle yield, into joint genetic evaluations is recommended to enhance market competitiveness (Berry et al. 2023; Coleman et al. 2023). Incorporating these traits into BoD evaluations would facilitate the identification of sires capable of producing calves with superior beef quality, aligning with the expanding BoD market. Furthermore, assigning a modest weighting to beef traits within dairy selection indices could effectively balance dairy production efficiency with enhanced calf market value, thereby improving overall farm profitability (Berry et al. 2020). In the present study, farmers selling calves to fattening farms prioritised BW for higher prices, making ANG and WBB sires the better candidates.

While EBVs for purebred beef cattle may exist for the traits considered in this study, BoD‐specific GEBVs are superior in predicting crossbred performance by accounting for interactions with dairy dams. A direct validation study comparing purebred EBVs to those involving a mixed population demonstrated accuracy improvements of 10%–20% based on prior multibreed analyses (Steyn et al. 2019). The limitations of purebred beef EBVs in predicting BoD performance stem from unaccounted breed interactions, underscoring the necessity of BoD‐specific evaluations (VanRaden et al. 2020). A head‐to‐head comparison of BoD‐specific GEBVs against purebred EBVs would quantify the benefits of crossbred evaluations, offering a promising avenue for future research.

The inclusion of crossbreds in the reference population enhances GEBV accuracy for BoD performance, as it captures heterosis and breed‐specific effects absent in purebred data (VanRaden et al. 2020; Wientjes et al. 2020). In the present study, the unequal representation of sire breeds, with Angus (ANG) and Wagyu‐cross (WBB) sires predominating, reflects farmer preferences for these breeds in BoD systems. To address breed differences, genetic evaluations for BoD must employ methodologies such as breed‐specific genomic relationship matrices (**G** or **H** matrices) (Makgahlela et al. 2014; Steyn et al. 2019) or models incorporating breed‐of‐origin allele effects (Eiríksson et al. 2021; Karaman et al. 2021). In this study, a joint **H** matrix approach, incorporating sire breed as a fixed effect and a random sire effect, effectively accounted for both between‐breed and within‐breed genetic variation.

### 4.4 Accuracy, Bias, and Dispersion

Estimates of accuracy, bias, and dispersion were obtained for both bivariate and univariate models. Accuracies for CE were lower than for GL and BW due to CE's lower heritability, as expected for binary traits (Legarra and Reverter 2018). Bivariate models outperformed univariate models, with CE accuracy increasing from 0.13 to 0.22 (Figure 5). The bivariate model's superiority is attributed to additional information from correlated traits (e.g., BW in BW‐CE), reducing bias (e.g., GL bias: 0.07 vs. 0.17) and improving dispersion (e.g., CE: 0.90 vs. 1.33). These results confirm the advantage of bivariate threshold models for CE when BW data are available (Varona et al. 1999; Ramirez‐Valverde et al. 2001; Vanderick et al. 2017). Higher accuracy indicates stronger associations between estimated and true breeding values, while bias and dispersion values closer to optimal (0 and 1) suggest adequate model shrinkage. A more balanced dataset (equal sire breed representation) could improve accuracy further, supporting precise sire selection for BoD systems.

## 5 Conclusions

From our study, ANG sires had the lowest probability for calving difficulty, but the resulting BoD calves were not as heavy as those from WBB. Farmers thus preferred heavier calves from WBB (to increase profitability) sires but from high parity cows (to minimise risk of difficult calvings). LIM sires had the highest probability of calving difficulty. Our results illustrate that the best strategy to select beef sires to inseminate dairy cows should consider the sire's breed, parity of the cow, and sex of the calf (when using sexed semen) to predict calving difficulty. Estimating those effects through the beef‐on‐dairy genetic evaluation helps to decide, for example, if WBB sires could be used in cows with 2, 3 or more parities at any given herd. Similarly, it will help decide when it is convenient to use LIM sires, minimising the risk of calving problems. With a more balanced dataset and more phenotypic records, a genetic evaluation of sires from different breeds can be refined to aid farmers in making informed decisions with more precision through better estimates of the effect included in the model.

## Conflicts of Interest

The authors declare no conflicts of interest.

## Supporting information


**Table S1:** showing descriptive statistics for the focal animals.

## Data Availability

The data that support the findings of this study is not publicly available. However, interested and qualified researchers may request access to the data by contacting the corresponding author. All data requests will require a formal data use agreement.
