# Supplementary material for: Genetic Evaluation of Beef Sires Using a Beef‐on‐Dairy Crossbred Reference Population
Source: J Anim Breed Genet. 2025 Nov 15;143(3):390–402. doi: 10.1111/jbg.70030 (PMC13054129; doi:10.1111/jbg.70030)
Supplement: Supplementary file 1 — Table S1: showing descriptive statistics for the focal animals. [file JBG-143-390-s001.docx]

| **Supplementary Table 1 showing descriptive statistics for the focal animals** | | | | | | |
| --- | --- | --- | --- | --- | --- | --- |
| **Sire Breed^1^** | **No. sires** | **No. BOD calves** | **CE** | | **BW/kg** | **GL/days** |
| **ANG** | 7 | 821 | 12.5 | 45.5 | | 280.2 |
| **LIM** | 5 | 177 | 13.0 | 45.4 | | 288.1 |
| **WAG** | 3 | 127 | 3.97 | 42.5 | | 282.8 |
| **WBB** | 16 | 2,861 | 11.1 | 49.6 | | 281.6 |
| ^1^ ANG = Angus; LIM = Limousin; WAG = Wagyu and WBB = Belgian Blue; GL= Gestation length (days); BW = age-adjusted birth weight (kg); CE = calving ease incidences (%) | | | | | | |
